# Elevation-dependent variations of tree growth and intrinsic water-use efficiency in Schrenk spruce (*Picea schrenkiana*) in the western Tianshan Mountains, China

**DOI:** 10.3389/fpls.2015.00309

**Published:** 2015-05-06

**Authors:** Guoju Wu, Xiaohong Liu, Tuo Chen, Guobao Xu, Wenzhi Wang, Xiaomin Zeng, Xuanwen Zhang

**Affiliations:** ^1^State Key Laboratory of Cryospheric Sciences, Cold and Arid Regions Environmental and Engineering Research Institute, Chinese Academy of SciencesLanzhou, China; ^2^College of Resources and Environment, University of the Chinese Academy of SciencesBeijing, China

**Keywords:** tree rings, basal area increment, carbon isotope, SPEI, elevation gradient, physiological response, Northwestern China

## Abstract

Rising atmospheric CO_2_ concentration (*C*_a_) is expected to accelerate tree growth by enhancing photosynthesis and increasing intrinsic water-use efficiency (iWUE). However, the extent of this effect on long-term iWUE and its interactions with climate remains unclear in trees along an elevation gradient. Therefore, we investigated the variation in the radial growth and iWUE of mature *Picea schrenkiana* trees located in the upper tree-line (A1: 2700 m a.s.l.), middle elevation (A2: 2400 m a.s.l.), and lower forest limit (A3: 2200 m a.s.l.), in relation to the rising *C*_a_ and changing climate in the Wusun Mountains of northwestern China, based on the basal area increment (BAI) and tree-ring δ^13^C chronologies from 1960 to 2010. We used the CRU TS3.22 dataset to analyze the general response of tree growth to interannual variability of regional climate, and found that BAI and δ^13^C are less sensitive to climate at A1 than at A2 and A3. The temporal trends of iWUE were calculated under three theoretical scenarios, as a baseline for interpreting the observed gas exchange at increasing *C*_a_. We found that iWUE increased by 12–32% from A1 to A3 over the last 50 years, and showed an elevation-dependent variation in physiological response. The significant negative relationship between BAI and iWUE at A2 and A3 showed that tree growth has been decreasing despite long-term increases in iWUE. However, BAI remained largely stable throughout the study period despite the strongest iWUE increase [at constant intercellular CO_2_ concentration (*C*_i_) before 1980] at A1. Our results indicate a drought-induced limitation of tree growth response to rising CO_2_ at lower elevations, and no apparent change in tree growth and diminished iWUE improvement since 1980 in the upper tree-line. This study may contradict the expectation that combined effects of elevated *C*_a_ and rising temperatures have increased forest productivity, especially in high-elevation forests.

## Introduction

Atmospheric changes, and particularly the recent human-induced increases in carbon dioxide (CO_2_) concentrations and temperature, significantly affect tree growth (Martinez-Vilalta et al., 2008; Allen et al., 2010; Koutavas, 2013). Sudden, rapid changes in the atmospheric CO_2_ concentration (*C*_a_) can dramatically affect short-term plant physiology and growth (Martinez-Vilalta et al., 2008), and research has shown that plants increase their intrinsic water-use efficiency (iWUE) in response to increasing CO_2_ (Morison, 1993; Morgan et al., 2004). Compare to these short-term experimental results, the long-term variations on the physiological responses to increased *C*_a_, obtained from the carbon isotope discrimination (Δ^13^C) of tree-ring series, give insight into how trees respond to increasing atmospheric CO_2_ concentrations under naturally growing conditions (Peñuelas et al., 2008, 2011). Some studies found that trees may vary in their response to increasing *C*_a_ as a result of interactions with other changing environmental factors, and especially with warmer conditions (Switsur and Waterhouse, 1998; and references therein; Fischlin et al., 2007). Recently, many studies focusing on changes in tree growth in response to rising atmospheric CO_2_ concentrations and climate warming have been carried out in natural stands (Peñuelas et al., 2008, 2011; Linares et al., 2009; Andreu-Hayles et al., 2011; Linares and Camarero, 2012a; Granda et al., 2014; Lévesque et al., 2014; Liu et al., 2014). Whether the increased iWUE in response to rising *C*_a_ enhanced tree growth varied among sites and species. The tree growth may potentially increase in response to rising *C*_a_ in moist temperate forests (Cole et al., 2010; McMahon et al., 2010), but often decreased in tropical, dry temperate forests and at the lower-elevation forest limit (Peñuelas et al., 2008; Nock et al., 2011; Lévesque et al., 2014). However, in the Mediterranean, *Juniperus thurifera* showed enhanced growth at increased iWUE despite unfavorable growing climate conditions (Granda et al., 2014), whereas riparian *Populus euphratica* showed a significant CO_2_-induced growth stimulation despite the negative influences of reductions in streamflow (Liu et al., 2014). However, there have been insufficient studies on the simultaneous elevation-dependent effects on tree growth in response to increasing *C*_a_ and climate warming.

The growth rate of plant species at northern latitudes and high elevations, near their range limit, correlates well with the mean or extreme temperatures (Körner, 2012; Lenz et al., 2014). This is an important effect, since most of the world has experienced an unequivocal warming trend in the past half century, and further warming is likely (IPCC, 2013). Therefore, an elevation-dependent growth response of trees to climate is expected. At high altitudes, global warming has potentially increased radial growth (Körner, 2012), as in the case of studies in the European Alps (Hartl-Meier et al., 2014b) and in North America (Salzer et al., 2009), but growth suppression is expected to occur at lower elevations due to the predicted increase in the frequency and intensity of drought (Weltzin et al., 2003; Peñuelas et al., 2008). The synergistic effect of elevated CO_2_ and temperature is expected to stimulate forest productivity in temperature-limited environments (Salzer et al., 2009), but may decrease growth in water-limited environments (Linares and Camarero, 2012b). Therefore, it is necessary to find ways to detect the growth variation that occurs along an elevation gradient in response to increased *C*_a_ and warmer temperatures, since the dominant climatic stressor for trees may change with increasing elevation (Hartl-Meier et al., 2014a,b). Measurement of various tree-ring parameters allows an assessment of potential changes in tree physiology and growth over time. The tree-ring stable carbon isotope (δ^13^C) record provides evidence of climate effects on stomatal conductance and of tree physiological responses to increased *C*_a_ over time, whereas the basal area increment (BAI) provides a reliable indicator of tree radial growth (Linares and Camarero, 2012b).

It has been found that warming-induced prolonged drought stress significantly contributed to the marked reduction of regional BAI in recent years at mid-altitude in Tianshan Mountains (Wu et al., 2013). However, an altitudinal survey of radial growth variation and the tree physiological responses to increased *C*_a_ from lower forest limit to upper tree-line are still unclear. In the present study, we therefore combined tree-ring δ^13^C and BAI to examine tree responses to rising *C*_a_ and climate warming along an elevational gradient in the Wusun Mountains of northwestern China. Our first goal was to determine whether the δ^13^C and BAI varied between sites on the three different elevations. Second goal was to identify the climate factors influencing the δ^13^C and BAI and to detect the pattern of increased iWUE in response to rising *C*_a_. Finally, we also want to detect whether the increases in iWUE could enhance growth and whether the tree growth in response to the increase in *C*_a_ and climate warming varied along an elevation gradient. Specifically, we hypothesized that (1) lower radial growth and high iWUE may be found at lower forest limit compared to upper tree-line, and (2) elevation-dependent growth variation may be due to the divergence of dominant climatic stressor and iWUE increase pattern.

## Materials and methods

### Study area and climate

**Figure 1:**
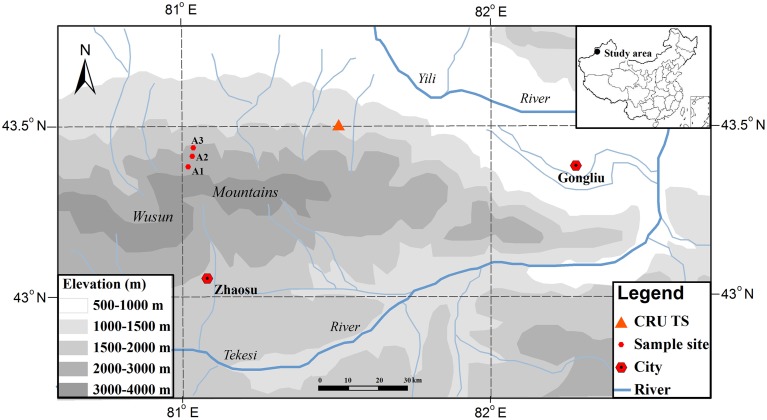
**Locations of the study area and of the three sampling sites and the nearest CRU TS location in the Wusun Mountains of northwestern China**.

The study area is located on the northern slopes of the Wusun Mountains in the Yili valley, which is located in the western Tianshan Mountains of northwestern China (Figure 1). This area is an intermontane basin that is open toward the west and surrounded by mountains on all other sides. Under these topographic conditions, the westerly airflow directly enters the valley, bringing moisture that produces precipitation as the air rises along the mountain slope; in contrast, the topography blocks dry and warm air flowing northward from the Tarim Basin and southward from the Junggar Basin, and cold air flowing from Siberia. The Yili Valley is dominated by a temperate semiarid continental climate (Zhu, 1985).

**Figure 2:**
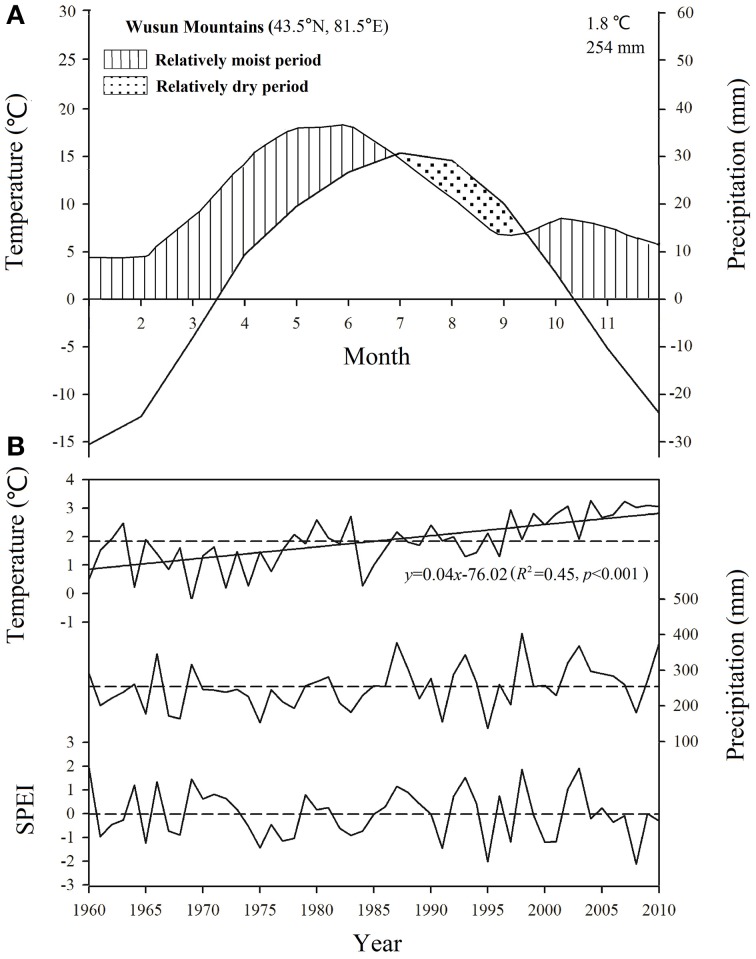
**(A)** Climate diagram based on data during 1960–2010 from the CRU TS3.22 dataset for the nearest point in the grid in the Wusun Mountains. **(B)** Trends in the mean annual temperature, total annual precipitation, and standardized precipitation evapotranspiration index (SPEI) in August based on a 5-month time scale for the study area from 1960 to 2010. Horizontal dashed lines represent the mean value for this period.

No site-specific climate data were available, therefore we used gridded climatic data from the CRU TS3.22 dataset, with a spatial resolution of 0.5 × 0.5° (Mitchell and Jones, 2005; http://www.cru.uea.ac.uk/). These data were derived from the nearest point in the CRU grid (43.5°N, 81.5°E). Hence, we only analyzed the general responses of tree growth to the inter-annual variability of regional climate in this study and our subsequent correlation analysis depended on changes in the values of each climate parameter (which should be similar along the altitudinal gradient) rather than on accurate values for each parameter. It is considered that even though the actual climate values (e.g., temperature, precipitation) will change along the altitudinal gradient, the patterns of change should be comparable along the altitudinal gradient (i.e., values should increase or decrease simultaneously at all altitudes) because they are mainly controlled by the broad-scale regional climate system. This view is supported by the results of two previous studies in the Tianshan Mountains (Guo et al., 2007; Wu et al., 2014). We used the monthly temperatures (mean, minimum, and maximum) and the monthly total precipitation from 1960 to 2010 in our analysis. The mean annual precipitation averaged 254 mm, with 62% of the precipitation falling from April to August, and the mean annual air temperature averaged 1.8°C, with the monthly mean ranging from -15.25°C in January to 15.4°C in July. The climate was dominated by a relatively dry period from July to September (Figure 2A).

To quantify the intensity of the water stress, we calculated the standardized precipitation evapotranspiration index (SPEI) using the SPEI Calculator software (http://digital.csic.es/handle/10261/10002) based on the CRU TS3.22 precipitation and temperature data. This index combines the ability to characterize evaporative demand at multiple scales with the ability to account for temperature variability (Vicente-Serrano et al., 2010). We calculated the SPEI value with a timescale of 5 months based on our subsequent correlation analysis, which showed that the climatic values during the 5-month period from April to August were most significant (based on data from 1960 to 2010) and considered the value in August to represent the maximum intensity of water stress during the growing season. The mean temperature showed a significant long-term increasing trend since 1960, whereas precipitation and SPEI showed no significant long-term trend (Figure 2B).

### Field sampling and stem radial growth at different elevations

Schrenk spruce (*Picea schrenkiana*) is one of the dominant species in the study area, and plays an important role in preventing soil erosion and soil water loss and in regulating climate, as well as in protecting the ecological stability of this inland river drainage area. Zhu et al. (2004) found that there were different relationships between the tree growth and climate at different aspects of slope and altitude of the valley. Schrenk spruce is an evergreen species that grows preferentially on shady (e.g., northwest-facing) slopes (Yuan and Li, 1995). It forms pure stands with a canopy cover ranging from 30 to 50%. The trees at our study sites grew on a northwest-facing slope (with an inclination of 30°) and the well-drained soil was a chestnut soil, which is equivalent to a Chernozems soil in the WRB soil taxonomy. We sampled this species near the upper (A1), middle (A2), and lower (A3) elevation limits of its distribution range (Figure 1; Table 1).

**Table 1:** **Site descriptions for the three sites used to obtain the three tree-ring chronologies**.

	**Site**		
	**A1-2700 m a.s.l**.	**A2-2400 m a.s.l**.	**A3-2200 m a.s.l**.
Dominant species	Schrenk spruce	Schrenk spruce	Schrenk spruce
Latitude	43°25′23.4″N	43°26′25.9″N	43°26′16.9″N
Longitude	81°02′36.7″E	81°05′02.6″E	81°05′32.7″E
Slope aspect	NW	NW	NW
Inclination (°)	30	30	30
Stand age (years)	123	99	95
Soil type	Chernozems	Chernozems	Chernozems
Soil depth (cm)	40	80–100	80–100
Sample size (no. of trees)	20	22	24

The number of sampled dominant healthy trees for dendroecological analysis were 20, 22, and 24 from A1 to A3. We collected two cores per tree at breast height, with the cores obtained at right angles to each other, from even-aged mature trees growing at the upper tree line, at a middle-elevation site, and at the lower limit of the forest. After air-drying and polishing the cores, we measured the ring width using the LINTAB 6.0 software (Rinn, 2003; http://www.rinntech.de) to a precision of 0.01 mm, and cross-dated the rings using the COFECHA software (Holmes, 1983). To account for the decrease in ring width that occurs with increasing tree size, we converted the radial increment into a BAI using the following formula (Phipps and Whiton, 1988; Biondi and Qeadan, 2008):

(1) $$BAI=\;\pi \times \left({\text{R}}_{n}^{\text{2}}-{\text{R}}_{n\text{-1}}^{\text{2}}\right)$$

where *R* is the tree radius and *n* is the year of tree-ring formation. The long-term variation of BAI was presented in the Supplementary Material (Supplementary Figure S1). In this study, we set the study period from 1960 to 2010 on account of the valid climate data acquisition and excluding the juvenile effects in the carbon analysis (McCarroll and Loader, 2004).

### Tree-ring δ^13^C and calculation of iWUE at different elevations

We selected five cores from different trees at each elevation that had the strongest correlation with the elevation-specific tree-ring chronology, few missing rings, and regular ring boundaries. We pooled the annual rings from the five samples by mixing all tree rings from a given year to produce a single composite sample, which we used to conduct the stable carbon isotope study. We extracted the α-cellulose using a modified version of the method of Green (1963) and Loader et al. (1997, 2008). To obtain highly homogenized α-cellulose, we used an ultrasound machine (JY92-2D, Scientz Industry, Ningbo, China) to break the cellulose fibers based on the method of Laumer et al. (2009).

The δ^13^C values were determined using a Flash EA1112 Elemental Analyzer coupled with a Finnigan Delta Plus mass spectrometer (Thermo Electron Corporation, Bremen, Germany) at the Key Laboratory of Western China's Environmental Systems, Lanzhou University. By convention, the carbon isotope ratio (δ^13^C) is expressed in delta (δ) notation with reference to the Vienna Pee Dee Belemnite (VPDB) standard, in parts per thousand (‰):

(2) $$\delta^{13}C=\left[\left({\text{R}}_{\text{sample}}/{\text{R}}_{\text{standard}}\right)-1\right]\times 1000$$

where *R*_sample_ and *R*_standard_ are the ^13^C/^12^C ratios in the sample and in the VPDB standard, respectively. During the experiment, laboratory reference standards were periodically used to calibrate the analytical results. The analytical error (standard deviation) of the isotope measurements was less than 0.07‰ for repeated samples.

The iWUE (μmol CO_2_ mol^−1^ H_2_O) compares the photosynthetic uptake of CO_2_ through the leaf stomata with the simultaneous transpirational loss of water through the stomata using the following formula (Farquhar et al., 1982):

(3) $$iWUE=\text{A}/{\text{g}}_{s}=C_{a}\times \left[\left(1-C_{\text{i}}/C_{\text{a}}\right)1.6\right]$$

where *A* is the rate of CO_2_ assimilation by the leaves, *g_s_* is the rate of leaf stomatal conductance, and *C*_i_ is the leaf intercellular CO_2_ concentration. To determine *C*_i_/*C*_a_, we used the equation proposed by Francey and Farquhar (1982):

(4) $$C_{i}=C_{a}\times \left[\left(\delta^{13}C_{\text{plant}}-\delta^{13}C_{\text{atm}}+1\right)/\left(b-a\right)\right]$$

where δ^13^C_plant_ and δ^13^C_atm_ refer to the δ^13^C composition of α-cellulose and of atmospheric CO_2_, respectively. The parameter *a* (4.4‰) represents the discrimination that occurs during diffusion of CO_2_ from the atmosphere into the intercellular space of cells, and *b* (27‰) is the assumed discrimination that occurs during carboxylation.

**Figure 3:**
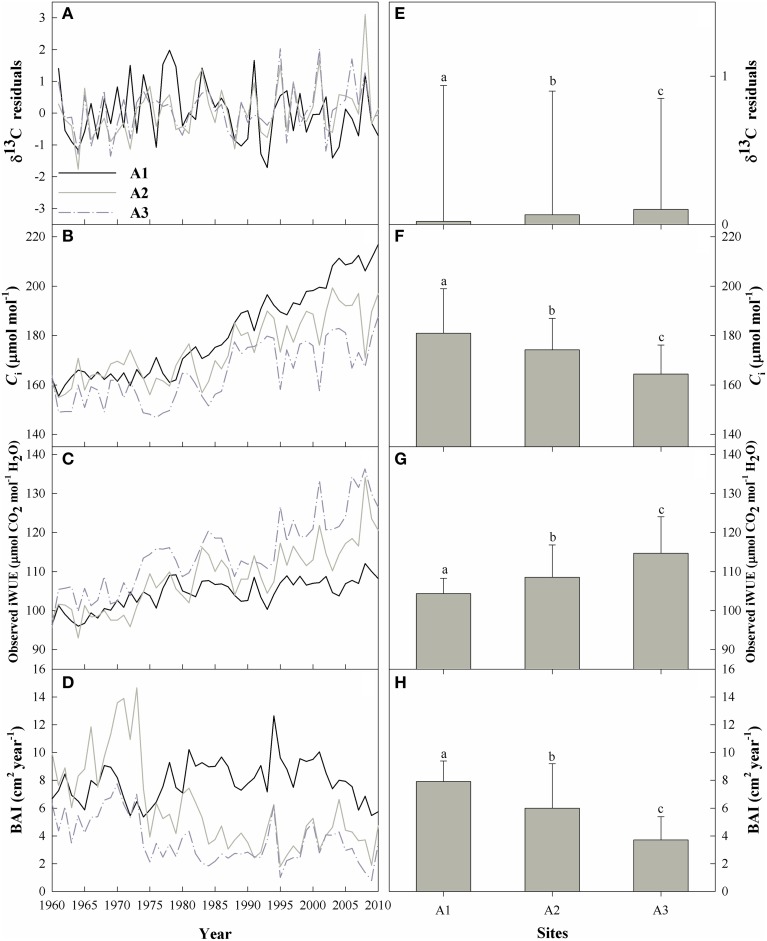
**Left: the temporal trends of (A) the δ^13^C residuals chronology, (B) the intercellular CO_2_ concentration (*C*_i_), (C) the intrinsic water-use efficiency (iWUE), and (D) the basal area increment (BAI) for the three sites from 1960 to 2010**. Right: the means and standard errors of **(E)** the δ^13^C residuals chronology, **(F)** *C*_i_, **(G)** iWUE, and **(H)** BAI for the three sites during the period from 1960 to 2010. Bars for a parameter that are labeled with different letters differ significantly between sites (*p* < 0.05, ANOVA).

However, tree-ring δ^13^C records are widely considered to show a prominent downward trend that can be attributed to the isotopically depleted CO_2_ in the atmosphere (δ^13^C_atm_) that has resulted from industrialization, and should be corrected for this effect by adding published annual values for δ^13^C_atm_ derived from ice cores as well as direct measurements during the modern era (McCarroll and Loader, 2004). The atmospheric δ^13^C_atm_ values from 2004 to 2010 were provided by Professor Danny McCarroll (Swansea University). Hereafter, we refer to this corrected data as the δ^13^C_cor_ data (δ^13^C_cor_ in Supplementary Figure S2). As a downward trend often remains even after correcting for changes in the atmospheric CO_2_ concentration as a result of changes in plant physiological responses to the increased *C*_a_ (Treydte et al., 2001; Gagen et al., 2007, 2011), we further corrected this data using the method proposed by McCarroll et al. (2009). Hereafter, we refer to this corrected data as the δ^13^C_pin_ data (δ^13^C_pin_ in Supplementary Figure S2). Due to autocorrelation in the δ^13^C_pin_ trend, we further standardized the δ^13^C_pin_series (we scaled the series to provide a mean of 0 and a variance of 1) and fitted an autoregressive model of order 1 to each series based on the method of Lévesque et al. (2013). The resulting δ^13^C residuals chronology was used in the following analysis (Figure 3A).

The tree-ring δ^13^C values reflect the plant's physiological response to climatic variables. Saurer et al. (2004) proposed three kinds of plant response to rising CO_2_: (1) *C*_a_ − *C*_i_ remains constant, *C*_i_/*C*_a_ increases, and iWUE remains constant (a passive response); (2) *C*_i_/*C*_a_ remains constant, but iWUE increases (an active response); and (3) *C*_i_ remains constant while iWUE increases strongly (strongest response). In this study, we used these three scenarios as a baseline for interpreting the observed the iWUE trends, based on the analysis described by Linares and Camarero (2012a) and Liu et al. (2014).

### Data treatment and statistical analysis

We used One-Way ANOVA to detect significant differences in the mean value of tree growth data among the three elevations using version 16.0 of the SPSS software (SPSS Inc., Chicago, IL, USA). We also performed correlation analysis to examine the relationships between the tree-ring data and the mean monthly and seasonal climate data. We calculated Pearson's correlation coefficient (*r*) between the δ^13^C residuals chronology, BAI, and the climate variables using SPSS. The relationships between BAI and iWUE were assessed using linear regression analysis via SPSS.

## Results

### Temporal and elevational trends

Overall, the means of the δ^13^C residuals chronology, *C*_i_, iWUE, and BAI differed significantly among the three elevations (Figure 3; Supplementary Table S1). The δ^13^C residuals chronology at the upper tree-line (A1) showed high δ^13^C enrichment before 1980 compared with the two lower-elevation sites (Figure 3A), but the chronology at the lower forest limit (A3) showed the highest δ^13^C for the study period as a whole (Supplementary Table S1). The δ^13^C enrichment was therefore significantly elevation-dependent (Figure 3E).

Mean *C*_i_ decreased with decreasing elevation (Figure 3F), with a mean value of 164.4 μmol mol^−1^ at site A3, vs. 180.9 and 174.2 μmol mol^−1^ at sites A1 and A2, respectively. During the study period, iWUE increased significantly with decreasing elevation (Figures 3C,G), and increased throughout the study period, with increases of 12.0, 23.0, and 32.0% at sites A1, A2, and A3, respectively (Figure 3C). iWUE remained relatively constant at site A1 after 1980, but increased continuously at sites A2 and A3 (Figure 3C).

BAI decreased during the past 50 years at sites A2 and A3, and the decrease was significant (*R*^2^ = 0.47 and 0.36, respectively; *p* < 0.0001), whereas BAI increased slightly but not significantly at A1 (Figure 3D). *C*_i_ and BAI were significantly lower and iWUE was significantly higher at the lower elevations (ANOVA, *p* < 0.01; Figures 3F–H).

### Climate–growth relationships

**Figure 4:**
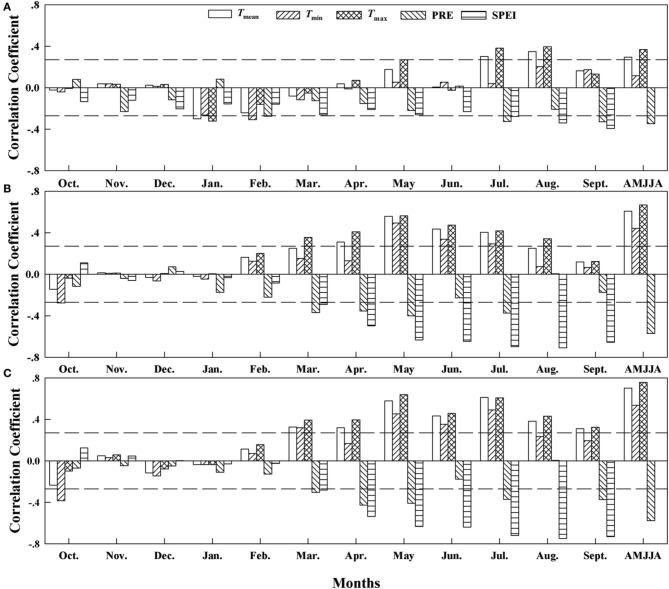
**Correlation coefficients (Pearson's *r*) between the δ^13^C residuals chronology and the climate variables from 1960 to 2010 at (A) Site A1 (2700 m a.s.l.), (B) Site A2 (2400 m a.s.l.), and (C) Site A3 (2200 m a.s.l.)**. Horizontal dashed lines represent the 95% confidence interval. *T*_mean_, mean monthly temperature; *T*_min_, minimum monthly temperature; *T*_max_, maximum monthly temperature; PRE, total precipitation; SPEI, standardized precipitation evapotranspiration index. AMJJA represents the mean value from April to August.

The correlation analysis based on the δ^13^C residuals chronologies (Figure 4) revealed significant correlations between the residuals and the mean temperature (*T*_mean_; *r* = 0.29, *p* < 0.05), maximum temperature (*T*_max_, *r* = 0.37, *p* < 0.01), and precipitation (PRE; *r* = −0.34, *p* < 0.01) from April to August at site A1. At sites A2 and A3, the correlations with SPEI (at A2, *r* = −0.71, *p* < 0.001; at A3, *r* = −0.74, *p* < 0.001) in August were stronger than those with temperature during the months from April to August. The strength of the correlation and the dominant climate variables that controlled carbon isotope discrimination therefore differed among the elevations.

**Figure 5:**
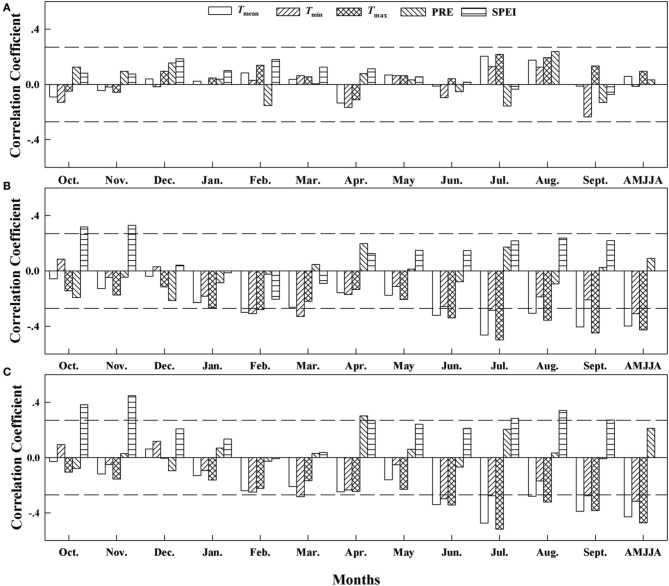
**Correlation coefficients (Pearson's *r*) between the response of the basal area increment (BAI) to the climatic variables based on data from 1960 to 2010 at (A) Site A1 (2700 m a.s.l.), (B) Site A2 (2400 m a.s.l.), and (C) Site A3 (2200 m a.s.l.)**. Horizontal dashed lines represent the 95% confidence interval. *T*_mean_, mean monthly temperature; *T*_min_, minimum monthly temperature; *T*_max_, maximum monthly temperature; PRE, total precipitation; SPEI, standardized precipitation evapotranspiration index

The climate-response patterns of BAI were also elevation-dependent, and the climate signal recorded in the BAI series was much stronger (*r* values were much higher) at lower elevations (sites A2 and A3) than at site A1 (Figure 5). BAI was not significantly correlated with any climate variable at the highest-elevation site. Thus, the temperature parameters during the growing season had a stronger and significantly negative relationship with BAI at the two lower elevations, and the strength of this correlation increased with decreasing elevation. The correlation between the temperature series from April to August and BAI was strongest at 2200 m (site A3: for *T*_mean_, *r* = −0.43, *p* < 0.01; for *T*_min_, *r* = −0.32, *p* < 0.01; and for *T*_max_, *r* = −0.47, *p* < 0.01). The correlations between BAI and precipitation were weak, with a significant correlation in April at site A3 (*r* = 0.30, *p* < 0.01), but we found an increasing correlation for SPEI with decreasing elevation; the correlation reached to *r* = 0.34 (*p* < 0.01) at site A3 in August with a timescale of 5.

The climate-growth relationship revealed significant climate signals in the tree-ring δ^13^C series but not in the BAI series at the highest-elevation site, but the impacts of temperature and SPEI on tree growth increased with decreasing elevation in both the δ^13^C analysis and the BAI analysis.

### Physiological responses of trees to increasing *C*_a_ at different elevations

**Figure 6:**
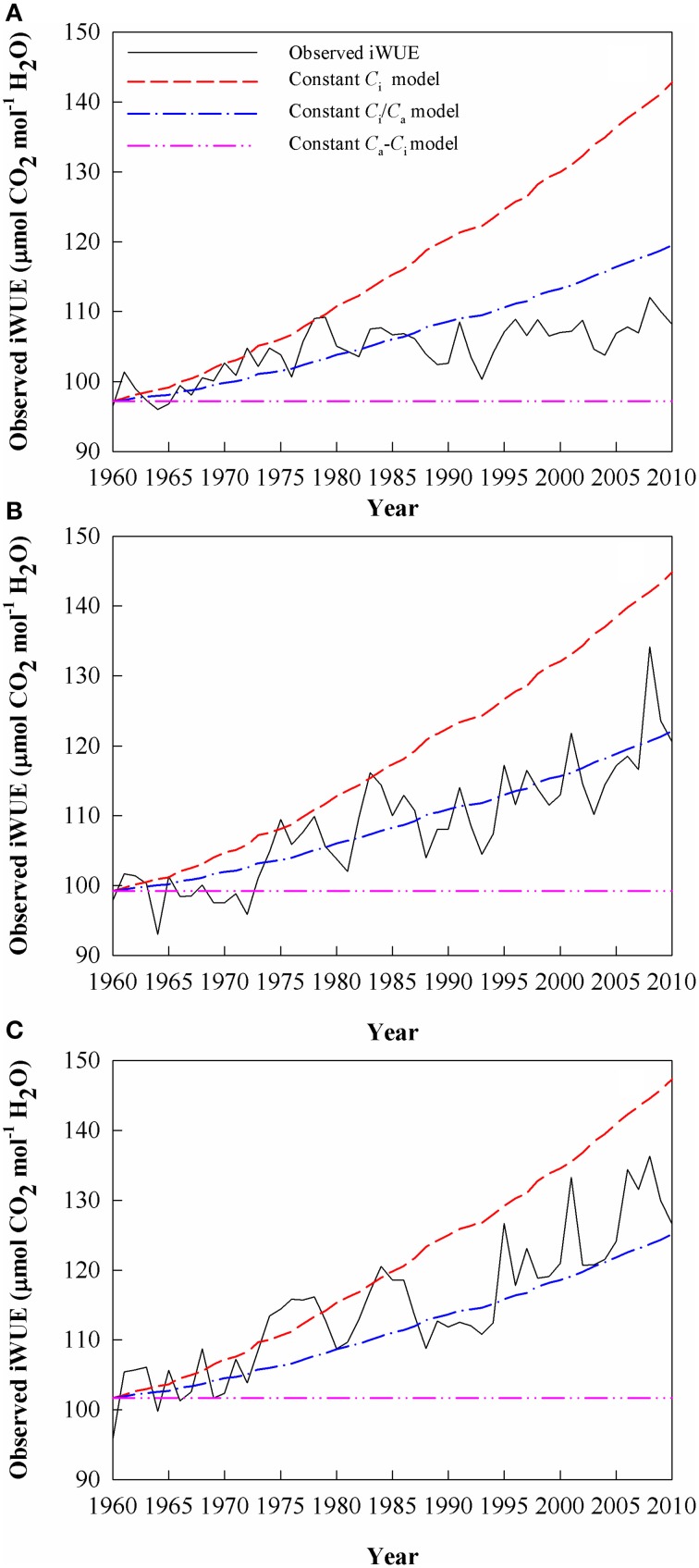
**The observed and theoretical changes in intrinsic water-use efficiency (iWUE) calculated using Equation (3)**. The three scenarios for the theoretical regulation of leaf gas exchange in response to an increasing atmospheric CO_2_mole fraction (*C*_a_) are: (i) a constant intercellular CO_2_ mole fraction (*C*_i_); (ii) a constant *C*_i_/*C*_a_ ratio, and (iii) a constant *C*_a_ - *C*_i_ value. Values are for **(A)** Site A1 (2700 m a.s.l.), **(B)** Site A2 (2400 m a.s.l.), and **(C)** Site A3 (2200 m a.s.l.).

Since 1960, iWUE at the highest-elevation site increased by 12.0% (by 0.23 μmol CO_2_ mol^−1^ H_2_O year^−1^), vs. increases of 24 and 32% (0.44 and 0.60 μmol CO_2_ mol^−1^ H_2_O year^−1^), respectively, at the middle-elevation and low-elevation sites (Figure 6). The dominant scenario that explains the iWUE trends differed among the sites and between two subsets of the study period (Figure 6). At the highest-elevation site, iWUE was dominated by constant *C*_i_ until 1980 (which assumes that is the strongest response to *C*_a_), with a 13.0% increase during this period, after which iWUE followed a pattern with constant *C*_a_ − C_i_, with no overall increase or decrease for iWUE from 1980 to 2010 (Figure 6A). However, the iWUE values at the mid-elevation site and at the lower forest limit were mainly dominated by a constant *C*_a_ − C_i_ scenario during the 1960s. Then, the iWUE increase is fallen within the range of expected iWUE values represented by the constant *C*_i_ and the constant *C*_i_/*C*_a_ scenarios at the mid-elevation and lower forest limit since 1973 (Figures 6B,C). After 1980, the iWUE trends at the mid-elevation site remained close to those in the constant *C*_i_/*C*_a_ scenario, but the contemporaneous iWUE values at the lower forest limit were higher than those predicted under a constant *C*_i_/*C*_a_ scenario.

### Relationships between radial growth and iWUE

**Figure 7:**
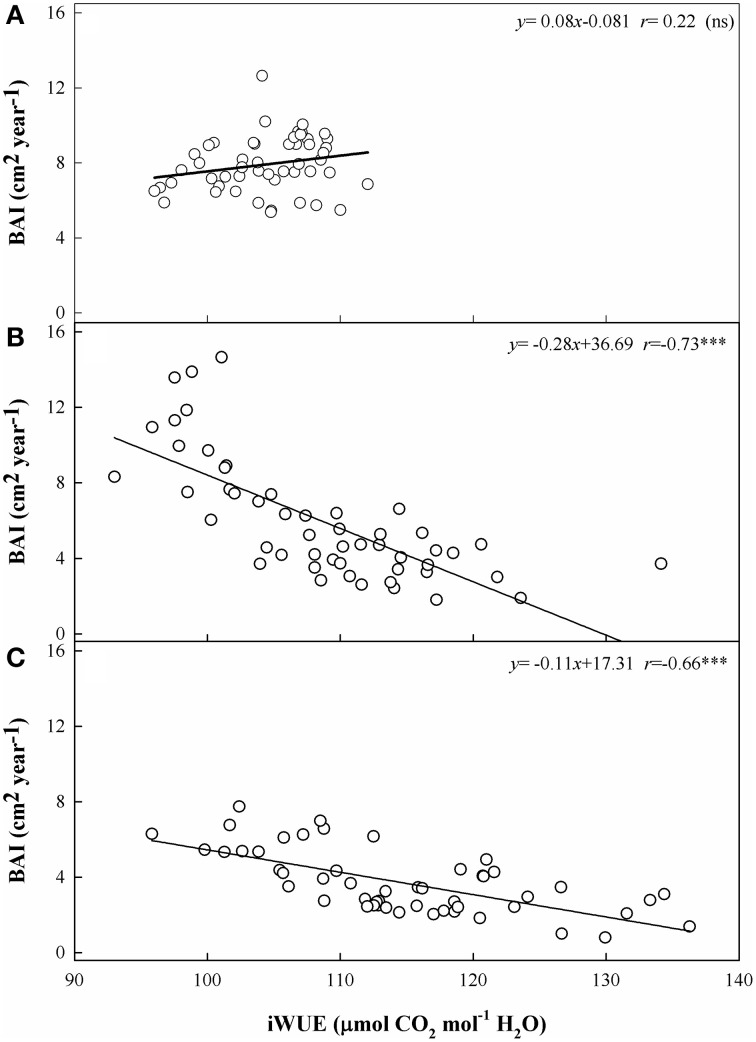
**Relationships between the basal area increment (BAI) and the intrinsic water use efficiency (iWUE) at the three sites from 1960 to 2010. (A)** Site A1 (2700 m a.s.l.), **(B)** Site A2 (2400 m a.s.l.), and **(C)** Site A3 (2200 m a.s.l.). Relationships between the two variables are calculated using Pearson's correlation coefficient (*r*). Significance levels: ^***^, *p* < 0.001; ns, not significant.

We detected the effects of long-term gas exchange on tree growth via the relationship between iWUE and BAI. BAI decreased significantly as iWUE increased at the low and middle elevation sites (*r* = −0.66 and *r* = −0.73, *p* < 0.001) (Figures 7B,C). In contrast, BAI at the upper tree-line remained stable (i.e., there was no statistically significant trend) despite increased iWUE (*r* = 0.22; Figure 7A).

## Discussion

### Temporal and elevation effects on the climate constraints

The weather conditions during the growing season (April to August) played a dominant role in determining the variation in tree-ring δ^13^C. Tree-ring δ^13^C is expected to be strongly affected by photosynthetic rates, which are governed both by temperature and by the photon flux at the upper tree line (McCarroll and Pawellek, 2001). The stronger moisture signal reflected in δ^13^C (Figure 4) at the low-elevation site (A3) than that at the highest-elevation site (A1) further indicates that trees suffered from enhanced water stress at A3. Moisture conditions, and specifically the vapor-pressure difference between the atmosphere and the intercellular air spaces of the leaves, directly determine the stomatal aperture, which in turn controls the leaf intercellular CO_2_ concentration (*C*_i_) and thereby governs δ^13^C (Farquhar et al., 1982).

The low and non-significant correlation (*r* < 0.276, *p* > 0.05) between the climate controls and BAI at site A1 suggests lower water stress and more adequate growth conditions for trees at the upper tree-line. In contrast with site A1, we found similar responses of tree growth to climate in trees growing at the A2 and A3 sites. Trees at both sites showed a similarly strong negative correlation with temperature throughout the growing season, but revealed increased sensitivity to moisture conditions (e.g., the strong negative correlations with SPEI; Figure 4) at the lower-elevation sites. High temperatures decrease soil water content and increase evaporation, hence reducing stomatal conductance, photosynthesis, and growth of trees (Peñuelas et al., 2008; Yin et al., 2008; Lévesque et al., 2013).

### Elevation-dependent physiological responses to the *C*_a_ increases

iWUE of Schrenk spruce has increased at all three sites since 1960 (by 12.0, 24.0, and 32.0% at sites A1, A2, and A3, respectively; Figure 6). These values fall within the range of iWUE increases (up to 36.2%) that has been reported in other forests (Bert et al., 1997; Duquesnay et al., 1998; Feng, 1998; Peñuelas et al., 2011; Linares and Camarero, 2012a). Nevertheless, some studies have found that the responses of trees to increased *C*_a_ differed between stands, even though the most pronounced changes in iWUE were observed during the second half of the twentieth century from tree-ring chronologies (Waterhouse et al., 2004; Andreu-Hayles et al., 2011; Gagen et al., 2011; Linares and Camarero, 2012a). We also found this change at the three sites in our study.

The highest-elevation site (A1) showed a significant decrease in *C*_i_/*C*_a_ before 1980, resulting in a greater iWUE improvement than at the other sites during this period, which agrees with the constant-*C*_i_ scenario (Saurer et al., 2004). Under this scenario, iWUE exceeded the expected values that would result from an active tree response to increased *C*_a_, suggesting that stomatal closure of trees at the highest-elevation site may be reinforced by other factors (Andreu-Hayles et al., 2011). The high elevation site has a lower atmospheric pressure, which may increase the effect of any increase in the partial pressure of CO_2_ on the rate of photosynthesis more than at lower elevations (Hultine and Marshall, 2000). In contrast, the A2 and A3 sites followed the constant *C*_a_ − C_i_ scenario during the 1960s, leading to the lowest iWUE increase during this period in response to increasing *C*_a_.

After 1980, we found a progressively smaller response to increasing *C*_a_ at the highest-elevation site. This lower sensitivity to *C*_a_ indicates that trees at the highest-elevation site may have become saturated with respect to increased *C*_a_, as the trees exhibited their greatest iWUE improvement from 1960 to 1980 at site A1 (Waterhouse et al., 2004; Figure 6A). However, the responses of the trees at the two lower-elevation sites were within the range of expected iWUE values under the constant *C*_i_ scenario and the constant *C*_i_/*C*_a_ scenario after 1973, a period when the greatest iWUE increases occurred at these sites. As the regional climate changes toward more aridification, generated by a significant temperature increase that was not accompanied by increased precipitation, hence increasing evaporative demand (Figure 2B), the greater iWUE improvement at lower elevations appears to have been induced by drought stress. We found that the limiting effects of drought for tree growth in lower elevation sites were strongly enhanced (Figures 4, 5). It is reasonable to expect a much lower water availability and enhanced drought stress at lower elevations, since the precipitation generally increases with altitudes in the Tianshan Mountains under the effect of westerly system (Guo et al., 2007; Wu et al., 2014).

### Tree growth at different elevations

The low-elevation site had consistently higher (less negative) δ^13^C_pin_, higher iWUE, and lower BAI than the A1 and A2 sites during the study period. Thus, the higher iWUE values since the 1970s that developed in response to the increased CO_2_ concentrations were consistently above those predicted under the constant *C*_i_/*C*_a_ scenario, but this was not sufficient to reverse the decrease in BAI at site A3 (Figure 7C), as was the case for the active-response scenario (constant *C*_i_/*C*_a_) at site A2 (Figures 6B, 7B). The significant negative relationship between tree growth (BAI) and iWUE at the A2 and A3 sites (Figures 7B,C), accompanied by the consistent increase in iWUE over time (Figures 6B,C), indicated that tree growth has been decreasing despite long-term increases in iWUE, especially at the lower-elevation sites. A trend of decreasing BAI is a valid indicator of a decline in tree growth (Phipps and Whiton, 1988), and does not appear to be a consequence of maturation of the trees (Leblanc et al., 1992). The decreasing BAI found at the lower-elevation sites is in line with recent studies that have demonstrated a warming-induced growth decline despite increasing iWUE for forest systems at dry sites (Peñuelas et al., 2008; Andreu-Hayles et al., 2011; Linares and Camarero, 2012a; Lévesque et al., 2014). Wu et al. (2013) have demonstrated that prolonged warming-induced drought stress contributed greatly to a marked reduction of the regional BAI in recent years for Schrenk spruce at middle altitudes on the northern slopes of the Tianshan Mountains. In the present study, the close relationship between BAI and SPEI at sites A2 and A3 further indicated a link between the growth decline and warming-induced drought at the lower-elevation sites (Figure 5).

However, at the upper limit of the current distribution of Schrenk spruce in the western Tianshan Mountains, BAI remained largely stable throughout the study period. Simultaneously, the changes in iWUE of Schrenk spruce at the A1 site agreed with the constant *C*_i_ scenario before 1980, with a particularly strong improvement in iWUE before 1980 (Saurer et al., 2004; Figure 6A). However, this iWUE improvement did not induce a significant overall increase in BAI (Figure 7A). After 1980, the lack of change in iWUE during a period with a constant *C*_a_ − C_i_ scenario suggests that the trees reached a limit in their ability to respond to the increased *C*_a_ and subsequently showed a diminishing sensitivity to rising *C*_a_. This diminishing sensitivity of iWUE and growth to increasing *C*_a_ at the tree line may be due to more adequate growth conditions at this site.

## Conclusions

In this study, we found a warmth-induced drought limitation on tree growth accompanied by increasing iWUE at the lower-elevation sites in the spruce forest. Moreover, the continuous increases in iWUE may not have been sufficient to counteract the decreases in tree growth that occurred at lower elevations, as the negative effects of other factors on tree growth, such as drought, may have outweighed the CO_2_ fertilization effect. In contrast, the strongest iWUE increase, which occurred before 1980, did not significantly increase tree growth at the upper tree line. After 1980, the sensitivity of iWUE to increasing *C*_a_ diminished, suggesting the existence of a saturation effect. Therefore, the synergistic effect of elevated CO_2_ and higher temperatures may have had different effects in temperature-limited environments (e.g., at the upper limit of the tree's distribution) than in water-limited environments (e.g., at the lower limit of the tree's distribution). However, this study showed no apparent change of tree growth and a diminishing of iWUE improvement since 1980 onward at upper tree-line, which may contradict expectations that combined effects of elevated *C*_a_ and rising temperatures have increased forest productivity, especially for the high elevation forest (Linares et al., 2009; Salzer et al., 2009). Our results may provide insights into the tradeoffs between the responses to increased *C*_a_ and climate change, which were revealed by changes in tree physiology (δ^13^C) and growth (BAI) along an elevation gradient in this semi-arid region of northwestern China.

## Author contributions

GW was responsible for the laboratory experiments and writing a draft of the manuscript. XL and TC contributed substantially to the conception or design of the work. GX, WW, XZ, and XZ contributed to the isotopic analyses. XL provided helpful suggestions and edits that improved the manuscript. TC, GX, WW, XZ, and XZ assisted in the statistical analysis and development of the chronologies. All the authors gave their final approval of the version to be published and agreed to be accountable for all aspects of the work by ensuring that questions related to the accuracy or integrity of all parts of the work will be appropriately investigated and resolved.

### Conflict of interest statement

The authors declare that the research was conducted in the absence of any commercial or financial relationships that could be construed as a potential conflict of interest.
