# Supplementary material for: Elevation-dependent variations of tree growth and intrinsic water-use efficiency in Schrenk spruce (Picea schrenkiana) in the western Tianshan Mountains, China
Source: Front Plant Sci. 2015 May 6;6:309. doi: 10.3389/fpls.2015.00309 (PMC4422019; doi:10.3389/fpls.2015.00309)
Supplement: Supplementary file 1 [file Table1.DOC]

***Supplementary Material***

**Elevation-dependent variations of tree growth and intrinsic water-use efficiency in Schrenk spruce (*Picea schrenkiana*) in the western Tianshan Mountains, China**

Guoju Wu ^1, 2^, Xiaohong Liu ^1 *^, Tuo Chen ^1^, Guobao Xu ^1^, Wenzhi Wang ^1, 2^, Xiaomin Zeng ^1, 2^, Xuanwen Zhang ^1, 2^

1. State Key Laboratory of Cryospheric Sciences, Cold and Arid Regions Environmental and Engineering Research Institute, Chinese Academy of Sciences, Lanzhou 730000, China

2. University of Chinese Academy of Sciences, Beijing 100049, China

*Corresponding author: Dr. Xiaohong Liu

State Key Laboratory of Cryospheric Sciences

Cold and Arid Regions Environmental and Engineering Research Institute

Chinese Academy of Sciences

No. 320 Donggang West Road

Lanzhou 730000, China

E-mail: liuxh@lzb.ac.cnTel.: 0086 93-1496-7342

Fax: 0086 93-1827-1124

**Supplementary Figures and Tables**

## 1.Supplementary Tables

**Supplementary Table S1. Summary statistics for the tree-ring δ^13^C_pin_ chronologies of Schrenk spruce at three elevations.**

|  | δ^13^C_pin_ (‰) | | |
| --- | --- | --- | --- |
|  | Site A1 (2700 m asl) | Site A2 (2400 m asl) | Site A3 (2200 m asl) |
| Mean | -22.07 | -21.59 | -21.04 |
| Maximum | -21.47 | -19.80 | -19.66 |
| Minimum | -22.57 | -22.79 | -22.49 |
| Range | 1.11 | 2.99 | 2.83 |
| Standard deviation | 0.25 | 0.55 | 0.62 |
| Variance | 0.061 | 0.30 | 0.39 |
| Skewness | 0.31 | 0.511 | 0.21 |
| Kurtosis | -0.14 | 1.132 | 0.031 |

## 2. Supplementary Figures

**
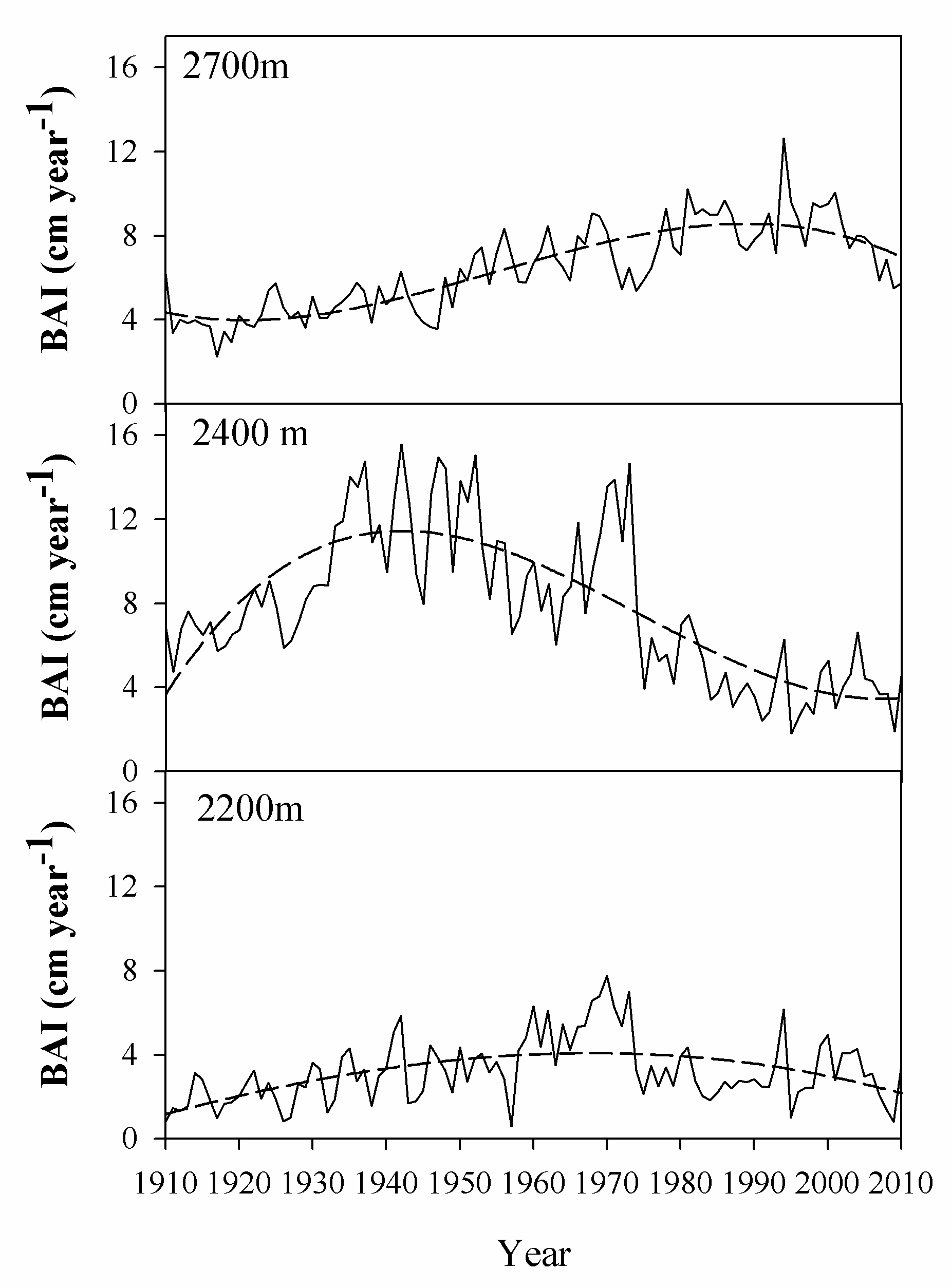
**

**Supplementary Figure S1.** The long-term variation of BAI at three elevation sites. The dash line represents a cubic polynomial regression.

**
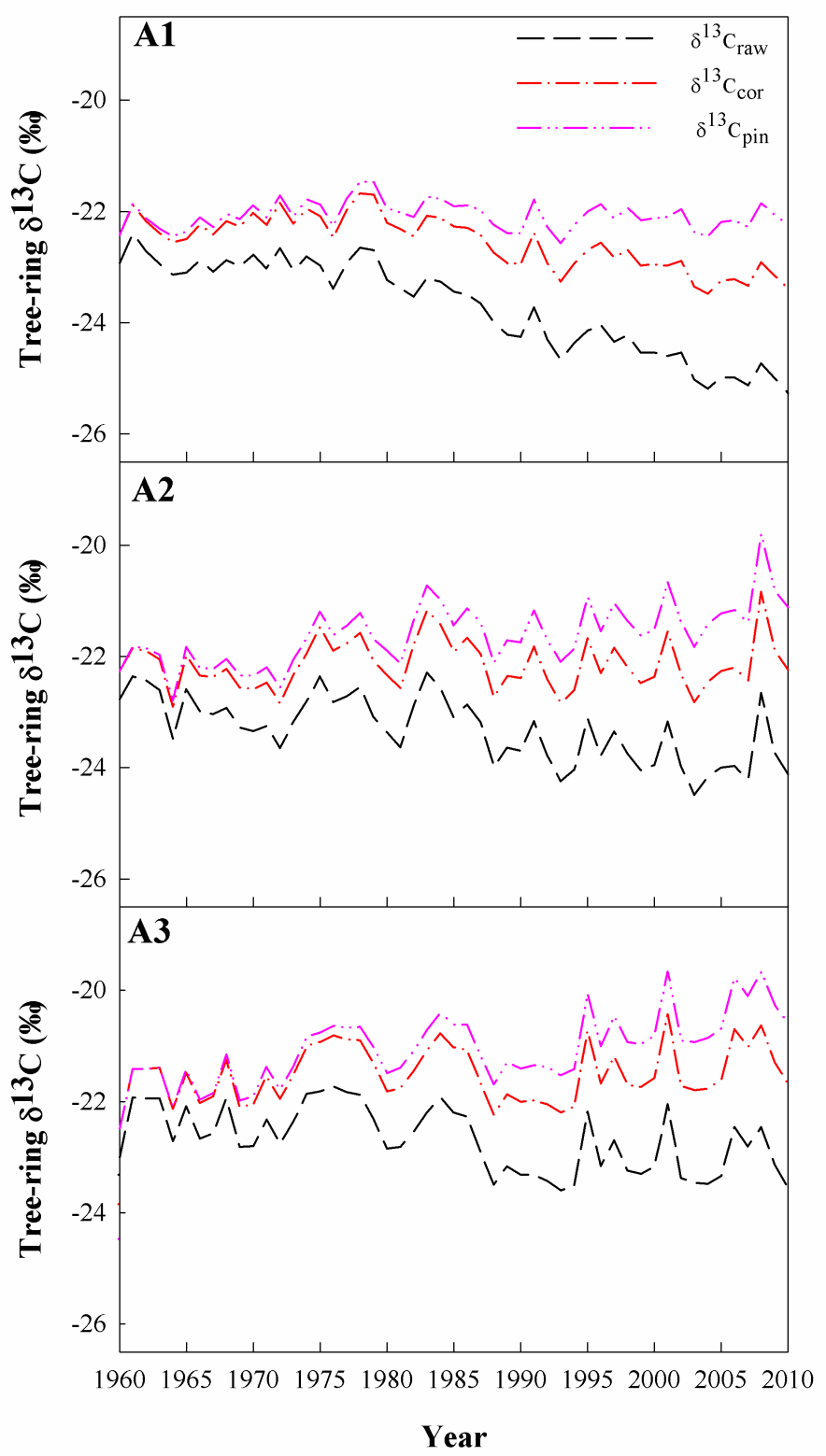
**

**Supplementary Figure S2.** The corrected tree-ring δ^13^C series at the three sites (A1, 2700 m a.s.l.; A2, 2400 m a.s.l.; A3, 2200 m a.s.l.) based on the measured data (δ^13^C_raw_), the corrected tree-ring δ^13^C_cor_ calculated using the method of *McCarroll & Loader* (2004), and the corrected tree-ring δ^13^C_pin_ calculated using the method of *McCarroll et al. (2009).*
